# Incidental Morgagni hernia found during laparoscopic repair of hiatal hernia: Case report & review of literature

**DOI:** 10.1016/j.ijscr.2019.03.012

**Published:** 2019-03-20

**Authors:** Ali Hassan, Marwah Alabdrabalmeer, Mohammad Alealiwi, Omran Al Danan, Saeed Alshomimi

**Affiliations:** a Department of Surgery, King Fahd Hospital of The University, Imam Abdulrahman bin Faisal University, Khobar, Saudi Arabia; b Department of Radiology, College of Medicine, Imam Abdulrahman bin Faisal University, Saudi Arabia

**Keywords:** Morgagni hernia, Hiatal hernia, Laparoscopic, Incidental

## Abstract

**Introduction:**

Morgagni hernia is rare clinical entity accounting for 3% of all surgically treated diaphragmatic hernias. Similarly, paraesophageal hernia constitutes only 5% of all hiatal hernia. The co-existing of these two hernias is extremely rare with only 10 cases reported in the literature.

**Presentation of case:**

We present a case of 53-year-old female patient with 6-year history of reflux disease. Her symptoms were poorly controlled by medications and she was seeking a surgical treatment. Preoperative assessment revealed a giant paraesophageal hernia for which a laparoscopic repair was planned. During the surgery, left-sided Morgagni hernia was discovered and both hernias were repaired at the same time. The patient tolerated the procedure well without complications.

**Conclusion:**

The co-existence of Morgagni and Hiatal hernia is rare and the simultaneously laparoscopic repair of both hernias is safe and feasible.

## 1 Introduction

Non-traumatic diaphragmatic hernia in adults is classified into late-onset congenital hernias (particularly, Bochdalek and Morgagni hernias) and hiatal hernia. Morgagni hernia is a rare entity accounting for 3% of all diaphragmatic hernias [1] and is commonly diagnosed and repaired during childhood. Hiatal hernia refers to the prolapse of abdominal cavity content (most commonly a portion of the stomach) through the diaphragmatic esophageal hiatus into the thoracic cavity. Paraesophageal hernia, which includes type II, III and IV, constitutes only 5% of all hiatal hernias [2].

The simultaneously occurring Morgagni hernia and paraesophageal hiatal hernia is extremely rare with only 10 cases reported in the English medical literature. Herein, we present a case of a patient who underwent laparoscopic repair of type III paraesophageal hernia and was found to have incidental left-sided Morgagni hernia. This case was written in line with SCARE Criteria [3].

## 2 Presentation of case

We present a 53-year-old female patient complaining of acid reflux and regurgitation for the last 6 years which was poorly controlled by medications and lifestyle modification. In the last few months, the symptoms became more severe despite the maximum medical therapy and her condition started to affect her quality of life. Hence, she was referred to our university hospital for further evaluation and possible surgical management. There was no history of dysphagia, odynophagia or weight loss. The past medical history is remarkable for intermittent bronchial asthma on Salbutamol inhaler when needed. The patient underwent previous laparoscopic cholecystectomy and Caesarean section. She had a body-mass-index (BMI) of 32 kg/m [2]. No history of smoking or alcohol drinking. Her physical examination was unremarkable for any abnormalities.

**Fig. 1:**
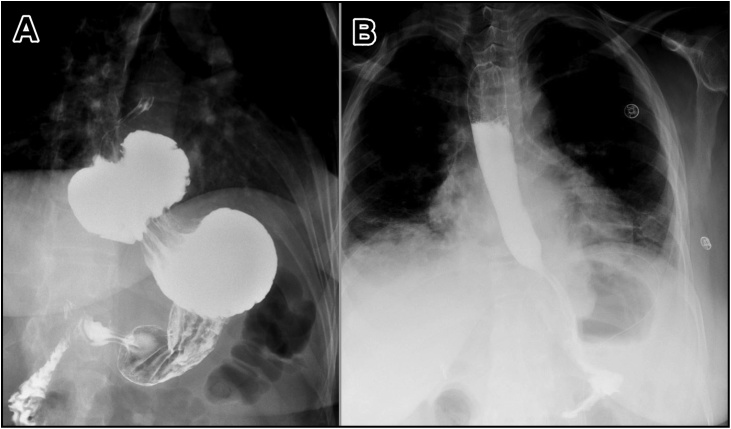
Barium swallow study pre-operatively showing the hiatal hernia (A) and post-operatively showing no evidence of leak with proper position of the stomach (B).

Pre-operative evaluations included upper gastrointestinal endoscopy, barium swallow and manometry. The endoscopy demonstrated an 8-cm hiatal hernia, grade B Los Angeles (LA) Classification of Reflux Disease and mild antral gastritis. The barium study confirmed the presence of hiatal hernia (Fig. 1A). The manometry showed normal esophageal motility. Upon these findings the patient was offered surgical treatment in the form of laparoscopic hiatal hernia repair with Nissen fundoplication, which the patient agreed for.

**Fig. 2:**
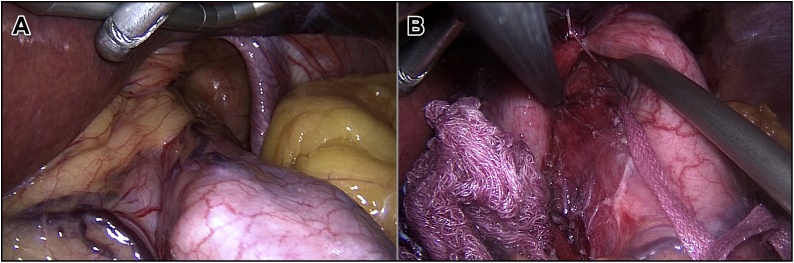
Laparoscopic view of the hiatal hernia in which the gastric fundus had invaginated into the hernia sac (A) and hernia defect after completing the repair (B).

**Fig. 3:**
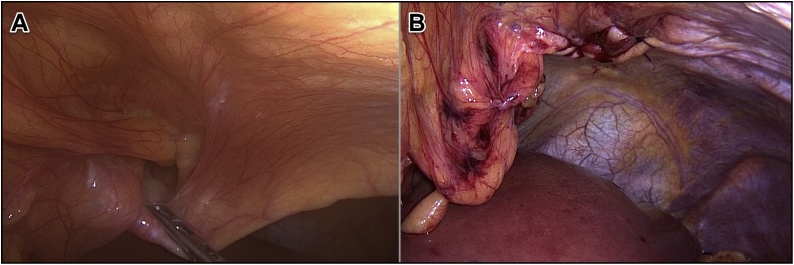
Laparoscopic view of the Morgagni hernia in which the falciform ligament had invaginated into the hernia sac (A) and hernia defect after the repair (B).

The operation was done under general anesthesia with the patient in a supine and leg-split position. Five ports were inserted; one 11 mm supraumbilical port for the camera, another 12 mm port in the left upper quadrant, two 5 mm ports inserted bilaterally at the midclavicular line at the level of umbilicus and lastly 5 mm port inserted below the xiphoid process. After establishing pneumoperitoneum and introducing trocars, standard diagnostic exploration was carried out. A giant hiatal hernia was noted with the gastric fundus herniated into the thoracic cavity (Fig. 2A). Furthermore, a small-sized left-sided Morgagni hernia containing a falciform ligament was seen incidentally (Fig. 3A).

The patient was placed in the reverse Trendelenburg position and snake liver retractor was used to expose the esophageal hiatus. The stomach was pulled back into the abdominal cavity and the peritoneal hernial sac was dissected and excised. Mobilization of the esophagus and gastric fundus was performed. Cruroplasty was done through interrupted sutures using Ethibond Sutures. A 360-degree Nissen fundoplication was carried out forming a wrap that measures about 2 cm in length (Fig. 2B).

The content of Morgagni hernia was reduced which was falciform ligament. The hernia sac was not resected. The sternocostal defect was measuring 3 cm in diameter. It was closed using continuous suturing of STRATAFIX™ 3.0 (Knotless Tissue Control Device – Ethicon, NJ, USA) and no mesh was used (Fig. 3B).

A 15-Fr BLAKE Silicon Drain (Ethicon, NJ, USA) was inserted through the left 5 mm port. The abdomen was deflated, ports were removed and incisions closed. The total operative time was about 120 min and blood loss less than 50 ml. The patient tolerated the procedure well.

In the first post-operative day, the patient had Gastrografin swallow and meal which demonstrated no evidence of leak with proper position of the stomach (Fig. 1B). The patient started on liquid diet and was tolerated. The patient stayed for 4 days post-operatively for pain control, consisting with our limited experience in Morgagni hernia repair – with or without use of mesh – that the post-operative course is often associated with severe pain necessitating longer hospital stay than in usual hiatal hernia repair. In the follow up visit, the patient had major satisfaction as her symptoms showed dramatic improvement after the surgery.

## 3 Discussion

Diaphragmatic hernia arises when there is a focal weakness or defect in the diaphragmatic musculature and a concomitant increase in the intra-abdominal pressure. Specific forms of such hernia include: Bochdalek hernia, Morgagni hernia, hiatal hernia and traumatic hernia.

The failure of fusion of the sternal and costal muscle fiber attachments of the diaphragm on either side of the xiphoid process leads to the formation of a potential site for herniation. The first case of such hernia was described in 1769 by the Italian anatomist and pathologist, Giovanni Battister Morgagni, while performing an autopsy [1]. The most common content of Morgagni hernia is omentum exclusively (34%) or colon and omentum (29%). Stomach, liver and small intestine may also be seen [4].

The majority of Morgagni hernia develops from the right sternocostal trigone. Left-sided Morgagni hernia, as in our case, is seen in 9% of all cases (5% isolated and 4% bilateral) [4]. Such rare occurrence is attributed to the protective effect of the pericardial sac [5].

Most cases of Morgagni hernia come to clinical attention during childhood. Contrary to the common belief that most cases in adults are asymptomatic, Horton et al. have shown that less than one third of patients (28%) have no symptoms at time of presentation and are discovered incidentally [4]. The average age of presentation in adult patients is 53 years [4]. The symptoms of Morgagni hernia are related to the content of hernia or the pressure exerted on the thoracic structures with common symptoms including pulmonary symptoms (e.g. dyspnea and persistent cough) and retrosternal chest pain; it rarely causes bleeding or intestinal obstruction [2,5].

Chest X-ray is the most common imaging modality used to evaluate patients with Morgagni hernia. It may show an intestinal gas pattern within the chest. The diagnostic test of choice is the computed tomography (CT) scan being the most sensitive and it may show the extent and the content of hernia [5]. Additionally, Morgagni hernia may be detected by upper gastrointestinal contrast studies if the sac contains parts of the stomach or intestine [1,6]. In our case, the chest X-ray did not reveal any abnormalities as the hernia was small in size.

Most surgeons agree that once the Morgagni hernia is diagnosed, prompt surgical repair becomes mandatory and there is no role of watchful waiting due to the risk of incarceration and strangulation of abdominal content in the chest [2,7].

Out of the various surgical approaches that can be used in the repair of Morgagni hernia, the transabdominal laparoscopic approach allows for easier reduction of the hernia sac, provides better exposure and visualization of the contralateral diaphragm as well as allows performing simultaneous procedures for other intra-abdominal pathologies. It is as effective as the traditional open approach with shorter hospital stay and higher patient satisfaction [4,8].

The defect of Morgagni hernia can be repaired primarily by approximation with non-absorbable sutures or by using plastic mesh to achieve tension-free repair. In our case, due to asymptomatic nature of the hernia and because of the small hernia defect size, we adopted the mesh-free repair method.

We chose to leave the hernia sac in-situ to avoid the risk of pneumomediastinum and injury to thoracic structures. This area is controversial as some surgeons adheres strictly to the principles of hernia repair stating that excision of the sac is essential as it decreases the fluid collection and reduces the tissue trauma, as the sac is manipulated instead of the content [4]. However, no such complications were noticed in our case.

In cases presenting with a single diaphragmatic hernia, the decrease in the intra-abdominal pressure might make the chance of a second diaphragmatic hernia formation less likely. However, our case along with the 10 cases reported in the literature for co-existing of Morgagni and paraesophageal hernia (Table 1) demonstrated that the contrary could occur. The etiology of co-existence is unclear and it is more likely a coincidental occurrence. The first reported case of simultaneous Morgagni and paraesophageal hernia was in 1958 by Lund et al. [6]. Upon review of clinical presentations of the reported cases, we found that all patients were symptomatic and in most cases, it was the paraesophageal hernia that caused the main symptomatic complaint. There was one case, similar to our case, in that a Morgagni hernia was found incidentally during the paraesophageal repair [9]. Furthermore, in the 9 out of 10 cases, Morgagni hernia was found on the right side. Different surgical approaches were used including conventional open (4 cases), laparoscopy (5 cases) and robotic-assisted surgery (1 case). Regarding the repair with mesh, it was used for both hernias in 2 cases while all other cases underwent direct repair without mesh use for either hernia.

**Table 1:** Simultaneously occurring Morgagni and paraesophageal hernias.

Case	Year	Authors	Age	Gender	Symptoms	Morgagni Hernia Side	Mesh Use		Content of Morgagni Sac	Surgery	Post-Op Hospital Stay	Complications
							Morgagni	Hiatal				
1	1958	Lund et al.	62	Female	Chest pain	Right	No	No	Transverse Colon	Laparotomy	NS	None
2	2001	Ngaage et al.	74	Male	Respiratory distress	Right	No	No	Omentum, Transverse Colon	Laparotomy	12 days	None
3	2002	Cokmez et al.	65	Female	Dyspnea, vomiting	Right	No	No	Omentum, Transverse Colon, Small Intestine	Laparoscopy	5 days	None
4	2003	Eroglue et al.	67	Male	Dyspnea, chest pain	Right	No	No	Omentum, Colon	Laparotomy	7 days	None
5	2006	Szentkereszty et al.	67	Female	Epigastric pain, dysphagia	Right	No	No	Omentum	Laparoscopy	5 days	None
6	2015	Bettini et al.	76	Male	Abdominal pain	Right	No	No	Distal Ileum, Cecum, Appendix, Ascending and Transverse Colon	Laparotomy	14 days	Pulmonary embolism, atrial fibrillation, C. difficile colitis
7	2015	Zhou et al.	73	Female	Chest pain, dyspnea	Right	No	No	Omentum	Laparoscopy	7 days	None
8	2018	Mittal et al.	71	Male	Regurgitation, heartburn, vomiting	Right	Yes	Yes	Omentum, Transverse Colon, Stomach	Laparoscopy	8 days	None
9	2018	Ozawa et al.	91	Female	Vomiting	Right	No	No	Omentum, Round Ligament	Laparoscopy	30 days	None
10	2018	Fu et al.	67	Female	Dyspnea, chest pain, back pain, dysphagia, heartburn	Left	Yes	Yes	Omentum	Robotic-Assisted	NS	None
11	Our Case		53	Female	Regurgitation, heartburn	Left	No	No	Falciform Ligament	Laparoscopy	4	None

## 4 Conclusion

Co-existence of paraesophageal and Morgagni hernia in adults is very rare especially left-sided ones. In addition, simultaneous laparoscopic repair for both hernias is a safe and feasible technique.

## Conflicts of interest

The authors declare that they have no competing interests.

## Sources of funding

This research did not receive any specific grant from funding agencies in the public, commercial or not-for-profit sectors.

## Ethical approval

No ethical approval needed; case report would be published without any identification data.

## Consent

Written informed consent was obtained from the patient for publication of this case report and accompanying images. A copy of the consent is available for review by the Editor-in-chief of this journal on request.

## Author contribution

Ali Hassan: drafting the manuscript; review of literature.

Marwah Alabdrabalmeer: editing and finalizing the manuscript.

Mohammad Alealiwi: obtained patient’s data and consent; review of literature.

Omran Al Danan: finalizing the manuscript.

Saeed Alshomimi: responsible surgeon; supervised the work; critically revised the manuscript.

## Registration of research studies

Not Applicable (Case Report; not an interventional study) and not needed by our institute.

## Guarantor

Dr. Saeed AlShomimi

Consultant Gastric Tumours and Upper GI Surgeon

King Fahd University Hospital, Imam Abdulrahman Bin Faisal University, Khobar, Saudi Arabia

## Provenance and peer review

Not commissioned externally peer reviewed.
